# The Commercial Determinants of Violence: Identifying Opportunities for Violence Prevention through a Public Health-Based Framework Analysis

**DOI:** 10.3390/ijerph21030352

**Published:** 2024-03-15

**Authors:** Mark A. Bellis, Sally McManus, Karen Hughes, Olumide Adisa, Kat Ford

**Affiliations:** 1 Public Health Institute, World Health Organization Collaborating Center for Violence Prevention, Faculty of Health, Liverpool John Moores University, Liverpool L2 2QP, UK; 2 Violence and Society Centre, City, University of London, London EC1V 0HB, UK; sally.mcmanus@city.ac.uk; 3 Policy and International Health, World Health Organization Collaborating Center on Investment for Health and Well-Being, Public Health Wales, Wrexham LL13 7YP, UK; karen.hughes18@wales.nhs.uk; 4 College of Medicine and Health, Bangor University, Wrexham LL13 7YP, UK; k.ford@bangor.ac.uk; 5 Institute of Social Justice and Crime, University of Suffolk, Ipswich IP4 1QJ, UK; o.adisa@uos.ac.uk

**Keywords:** commercial determinants, violence, marketing, employment, firearms, alcohol, pollution, climate

## Abstract

Violence has immediate and long-term repercussions for the health of individuals and communities. Recent increases in the understanding of public health approaches to violence prevention have focused on the policies and practices of government, health, and other public sector agencies. However, the roles of commercial bodies in fostering and preventing violence remain largely unaddressed. The wealth and influence of some companies now exceeds that of many countries. Consequently, it is timely to explore the roles of commercial processes in violence. Using a conceptual framework for the commercial determinants of health, we examine seven practices: political; scientific; marketing; supply chain and waste; labor and employment; financial; and reputational management. We include areas directly linked with violence (e.g., firearms) and those that indirectly impact violence through the following: design and promotion of products; employment practices; and impacts on environment, poverty, and local resources. A range of avoidable commercial behaviors are found to increase levels of violence including the following: lobbying practices; distortion of scientific processes; polluting manufacture and supply lines; poor employee protections; financial investment in organizations and regimes associated with violence; and misleading communications and marketing. We conclude commercial actors can take action to ensure their workers, clients, suppliers, and distributors help prevent, not promote, violence. New technologies such as artificial intelligence are transforming corporate processes and products and offer opportunities to implement violence prevention through commercial developments (e.g., monitoring online content). International regulation of commercial behaviors is needed to prevent interpersonal and interstate conflict and harms to health and trade.

## 1. Introduction

Globally, violence is an issue of epidemic proportions. Annually, half of all children in the world experience violence [1] and one in seven ever-partnered women (aged 15–49 years) are physically or sexually assaulted by an intimate partner [2]. Other aspects of interpersonal violence are major causes of harm to men. Thus, violence is the second leading cause of death in men aged 15–29 years with many more cases of ill-health and disability also resulting from violence [3]. Work on the commercial determinants of health encourages the adaptation of frameworks to understand opportunities for better well-being [4]. Despite the pervasive natures of violence and commerce, links between them are often obscured. Here, we apply a commercial determinants framework to violence. 

Violence can occur between individuals, groups such as gangs and militias, and factions within countries and across country borders. Whilst war is often considered separate from individual and community violent events, there are strong connections between them. Post-war settings frequently experience high levels of interpersonal violence [5], whilst such violence, especially between different ethnic, religious, or other groups, increases the chances of conflict [6]. Thus, conflicts and even individual acts of violence can be ‘infectious’, resulting in further violence elsewhere. Violence can degrade and destroy commercial interests in communities, countries, and even regions, adversely impacting economic stability, the protection of business assets, labor opportunities, and the reliability of supply lines [7]. However, the risks of violence are also impacted by commercial behaviors, depending on the values companies adopt and the influences they exert on legislation and regulation.

There has been substantial growth in the understanding of risk and protective factors for violence at individual, relationship, community, and societal levels [8]. Such evidence continues to develop and underpins public health strategies for violence prevention nationally and internationally [9]. However, like many public health issues [10], violence problems and solutions have been framed in ways that obscure the role of commercial actors. Commercial impacts may be direct or indirect, with public sector decisions influenced by private sector preferences as government policy adapts to commercial lobbying, sponsorship, and investment deals subject to conditions dictated by corporations [11]. The growing dominance of neoliberalist polices that favor capitalist freedoms with limited democratic control has contributed to the wealth and influence of major companies [11], with the richest companies’ values exceeding the Gross Domestic Product (GDP) of even some high-income countries [12]. Consequently, a traditional focus on policy and public sector reform fails to recognize the growing role that commercial organizations play in shaping public health and safety. For violence, its causes and opportunities for its prevention typically focus on the behaviors of individuals and responses from the public sector with little or no attention given to commercial determinants.

Incorporating commercial determinants into the consideration of drivers of public health requires a broad approach which includes biological, social, environmental, and behavioral issues as well as financial and other corporate considerations. Such a transdisciplinary approach is consistent with the objectives of this journal [13], whilst the multi-national nature of many commercial issues aligns with its global health theme. Consequently, here, we use a conceptual framework developed to classify commercial practices that affect health as a starting point for mapping the potential commercial determinants of violence. Our aim is to apply such a framework [11] to consider its utility for identifying risk and protective factors for violence, existing good practices, challenges, and opportunities for violence prevention.

## 2. Methods

Gilmore and colleagues [11] proposed a conceptual framework for the commercial determinants of health that grouped practices into seven areas: political; scientific; marketing; supply chain and waste; labor and employment; financial; and reputational management (Table 1). The framework recognizes overlaps between these practices, and with marketing and reputational management particularly interlinked; we consider these together. Other links between practices are identified where appropriate and summarized in the discussion. We examine a series of for-profit industries, of which most have no apparent direct links with violence but affect risks of violence through the design, use or misuse, and promotion of their products; their employment practices; and their impacts on environment, culture, poverty, and resources. Whilst recognizing that small- and medium-sized businesses account for a substantial amount of global commerce, we focus primarily on larger transnational companies that increasingly dominate commercial activities and influence political landscapes. We use the term commercial actors to incorporate the wide range of companies, corporations, and trading structures that collectively deliver commercial activities. 

We draw on examples from industries including alcohol, firearms, pharmaceuticals, and information technologies but do not exclude any industry and recognize that different aspects of business work in an integrated fashion. Examples are used to illustrate the ways in which commercial practices can influence the nature and extent of violence in society and to consider potential responses from individuals, commercial actors, and national and international governance. The academic literature on commercial determinants of violence is at an emergent stage; thus, we used a non-systematic scoping review approach combining searches of academic databases (e.g., PubMed), international organizations (e.g., World Bank), and the internet to identify relevant evidence from peer-reviewed journals, grey literature, and media sources. All sources were checked by at least two authors for credibility. 

The World Health Organization (WHO) defines violence as the intentional use of physical force or power, threatened or actual, against oneself, another person, or against a group or community, that either results in or has a high likelihood of resulting in injury, death, psychological harm, maldevelopment, or deprivation [8]. Their typology of interpersonal violence identifies four forms: physical, sexual, psychological, and deprivation or neglect. Threats, bullying, and coercion are included in this understanding, and settings can span any location (domestic, public, work, education, and institutional) as well as contexts of war, migration, repressive regimes, and international waters. Our focus is primarily on interpersonal violence. However, forms of collective, cultural, and structural violence are considered where relevant. Given recent examination of the commercial determinants of self-directed violence [14], these are not included.

## 3. Framework Application

### 3.1. Political Practices

The political practices of commercial actors range from the global to the local and include the following: direct and third-party lobbying; political party sponsorship; producing and using misleading information; threatening and taking legal action; and intimidating opponents [11]. Lobbying often frames problems as resulting from the behavior of individuals rather than the environment created by a lack of appropriate commercial regulation. When regulation is mooted, it may be countered with offers of self-regulation, potentially maintaining conditions conducive to violence. Thus, by lobbying against gun control regulations, firearm industries maintain high public accessibility to guns, increasing risks of violence, including homicide [15]. The alcohol industry lobbies against regulations that would limit profits and sales through, for instance, control of advertising, pricing, taxation, and age restrictions [16]. However, increased alcohol consumption is strongly associated with violence [17] and alcohol regulation has been linked to reduced interpersonal violence [18]. Pharmaceutical companies lobbying against drug restrictions contributed to the over-prescription of opioids in North America [19]. Opioid use has direct links with violent behavior including intimate partner violence [20] and may be linked to drug-related crime and violence [21,22]. Equally, the gambling industry actively resists legislative measures designed to reduce gambling-related harms despite links with intimate partner, child, and other violence [23,24]. Although evidence on the impact of video gaming content on players’ aggression is contested [25], industries reject considerations that restrictions on highly violent content may be prudent [26]. 

Cyberbullying and violence-inciting content are common features on social media platforms owned by multi-billion-dollar tech companies [27]. However, such companies have lobbied against legislation that would increase their accountability for content [28]. More widely, by funding or directly lobbying against environmental regulations [29], industries can exacerbate environmental degradation, leading to increased risks of violence (see Section 3.4). Revolving door practices facilitate lobbying as legislators or regulators move into employment in major companies and vice versa.

### 3.2. Scientific Practices

Commercial actors can influence scientific processes through mechanisms such as the following: funding research favoring their own interests; attempting to influence scientific editorial decisions; engaging scientists to publish ghost-written articles; stifling or concealing scientific developments; influencing governments in setting research agendas; and personally attacking or buying-out leading researchers in fields of interest [30,31]. For instance, lobbying by the US National Rifle Association (NRA—a recipient of firearms industry funding [32]) led to a legislative provision (the Dickey Amendment) prohibiting US Centers for Disease Control and Prevention from addressing gun control, deterring their involvement in research on firearms violence [33]. The alcohol industry has sponsored research which downplays alcohol’s negative effects, with the potential to encourage consumption and increase risks of violence [34]. The petroleum industry was aware of the trajectory of global warming in the 1970s and was advised that the continuation of then-current fossil fuel production trends would likely lead to ‘globally catastrophic effects’ by the 2060s [35]. Despite this, evidence was suppressed, and commercial activities continued. Climate change and rising temperatures have been found to increase violence, including collective, sexual, and domestic violence [36,37]. 

Large philanthropic foundations, often linked to major commercial actors, have invested in research directly and indirectly related to violence prevention [38]. Such research can beneficially contribute to understanding violence and its prevention. However, its context, content, and communication may be determined by funders, and it rarely addresses corporate culpability. Moreover, such investments can detract attention from actions linked with violence. For example, Silicon Valley technology companies have been accused of developing surveillance and military applications linked with human rights abuses [4].

Raising public awareness of the science of violence and its prevention can be important in effecting change. Between scientific research and its political and public consumption are communication industries that summarize and contextualize research findings. Such media are often part of wider news corporations, other businesses (e.g., IT companies), or bespoke science publishers that operate without formal peer review and may be controlled by larger conglomerates or reliant on advertising income and sponsorship. Mass media may also absolve corporations and governments, diverting attention away from regulation and towards individual-focused interventions [11]. The editorial decisions, sponsored content, and appetite to cover science linking violence to commercial determinants requires further scrutiny.

### 3.3. Marketing Practices and Reputational Management

Practices designed to promote product or service sales can perpetuate violent norms or related behaviors. Purdue Pharma aggressively promoted the prescription of Oxycontin to vulnerable potential patients, leading to addiction and increased risks of violence [39,40]. Firearms and alcohol industries have been criticized for designing and marketing products (e.g., weapons and alcoholic drinks) to appeal to youth [41,42]. Alcohol retail outlets use discounts, multi-buy deals, and other promotion strategies to encourage people to drink more [43], with implications for public and domestic violence [44]. Industry-funded youth education programs and teaching materials can promote alcohol consumption and provide a narrowed perspective on its harms—omitting evidence-based approaches to harm prevention [45]. Parts of the alcohol industry provide public messages against underage drinking, excessive consumption, and inappropriate alcohol promotion (e.g., Drinkaware, UK). Whilst ostensibly beneficial for violence prevention, such messaging can be poorly promoted compared to consumption-promoting advertising and used by corporations to resist more effective regulation [46]. 

Social media platforms have been criticized for effectively marketing violence [47], facilitating abuse, and normalizing discriminatory and abusive cultures (e.g., extreme misogynism [48]). Their practices have also been implicated in the radicalization and recruitment of violent extremists [49]. Age controls for social media and other online platforms containing explicit content, including music and videos, are often absent or easily circumvented by minors. Many scientific studies have shown that the mere presence of guns can increase aggression (the weapons effect). This effect has been examined from the perspective of film and other media with multiple studies linking exposure to media violence with increased aggression, particularly in children. The introduction of film age-based rating systems, whilst potentially beneficial to the reputation of the coordinating film industries, has been accompanied by increases in the explicitness of violence and depiction of guns in violent scenes [50]. Exposure to violent music lyrics has also been linked to increased anger and aggressiveness [51]. Equally, however, prosocial lyrics may improve empathy and mood [52]. Such effects have been explored in therapeutic settings but the potential contribution of prosocial music and videos to broader violence prevention requires greater study. 

Sport is a global industry often positioned as developing citizenship, tolerance, and prosocial behavior, which may support violence reduction. Empirical evidence for such benefits arising from sports engagement is more equivocal; although, some sports and exercise (e.g., yoga [53]) may reduce individual aggression. Larger sports businesses (e.g., football) have co-developed various violence-reducing measures (e.g., consensual policing, crowd control, and match-day management) [54]. However, these measures are required, in part, because of tribalistic aggression—sometimes deliberately instilled by competitive sport and promoted through sports teams and media companies [55]. Moreover, commercial sports, brands, events, and teams have developed relationships with countries with poor human rights records and norms supportive of violence, including against women and minority groups. Examples include Qatar’s hosting of the FIFA 2020 World Cup and Saudi Arabia’s LIV golf tournament [56,57]. Such relationships may stifle pressures for nations to enact violence prevention measures and create an impression, through links to popular sports teams, that violent practices are acceptable; or divert media attention from the abusive nature of some regimes to a palatable veneer of philanthropy [58]. Commercial sports companies also accept sponsorship from industries (e.g., alcohol [59]) where regulation could reduce violence. Often termed ‘sports washing’ [58], the improved public image that industries recoup for sponsorship deflects public attention from harms associated with their products and practices, increasing their ability to evade governmental regulation. For instance, Brazil banned alcohol in football stadiums in 2003 to reduce violence yet was pressurized by FIFA and the alcohol industry to remove this ban as part of hosting arrangements for the 2014 World Cup [60]. Televised sports events on commercial TV channels routinely carry adverts for alcohol and gambling products [61,62], despite such events attracting child audiences and the products being linked with violence [44,63]. 

Such practices are part of increasingly sophisticated reputational management aimed at shaping legitimacy and credibility and enhancing corporate brand image. Frequently, third-party companies, often international, may be utilized to advise on strategies or influence social media content and communications to promote products and influence public opinion. For example, the NRA evokes the US second amendment to place itself as a protector of the American public’s gun rights and routinely uses fearmongering to deter public support for gun regulation [64,65]; recently, US firearms industries have attempted to inhibit regulation directly through trade organizations [66].

### 3.4. Supply Chain and Waste Practices

Gilmore and colleagues [11] identify practices that are involved in the creation, distribution, retail, and waste management of products or services where corporations negatively affect human and planetary health. Such industries include agriculture, mining, and other industrial production that threaten local customs, communities, and resources. Violence arises where corporate activities include the intimidation and abuse of local communities and activists. For instance, a study examining violence associated with Canadian mining corporations in Latin America corroborated 44 deaths and over 400 injuries between 2000 and 2015 [67]. Between 2012 and 2022, Global Witness [68] recorded killings of almost 2000 environmental defenders, often linked to industries such as mining, agrobusiness, and logging; a third of those killed were from indigenous communities. Mining of rare materials (e.g., those used in modern electronics) can be undertaken by para-military organizations with consequences including increased interpersonal violence and extension of wars when activities occur in conflict zones. Such illicit activities ultimately rely on the sale and subsequent use of illegally mined minerals by legitimate electronics producers [69] who may choose to ignore the violence and human rights abuses endemic in their rare metal supply chains.

Exposure to industrial pollutants has been directly linked to increasing violent tendencies (e.g., lead [70]); even short-term air pollution exposure has been associated with increased violent crime [71]. Environmental exploitation and its subsequent degradation also lead to violence as irresponsible waste and pollution management practices, water diversion, cash crops, and climate change impact local communities. Multiple violent conflicts have arisen over access to water supplies diminished through industrial pollution, diversion (e.g., dams), and global warming [72]. Whilst modern inter-nation conflicts are not yet openly seen over, for instance, water, they have resulted in military posturing (e.g., Ethiopia, Egypt, and Sudan [73]). Some companies have recognized the fragility of watersheds and, at least to some extent, invested in their restoration [74]. Food scarcity is a recurrent historical theme in the causes of conflict and war and is likely to increase across the world due to global warming [75]. Governments have a key role in controlling global warming and industrial environmental degradation. However, investment decisions by commercial actors that align activity with shorter-term profit rather than sustainability exacerbate risks of violence.

Commercial actors adopt and promote their Environmental, Social, and Governance (ESG) credentials [76]. Partly synonymous with Corporate Social Responsibility (CSR), these showcase companies’ alignment with sustainable and ethical practices, and include environmental sustainability, respect for human rights, good citizenship, and workforce conditions, which can impact violence. Commercial actors use them for marketing practices and reputational management (Section 3.3), although this does not necessarily exclude them from reducing violence. How reliably ESG and CSR reflect genuine changes in commercial behaviors that impact violence requires further study. Thus, the United Nations Global Compact, the world’s largest corporate sustainability initiative which calls for companies to align strategies and operations with human rights, labor, environmental, and anti-corruption goals, had little impact [11,77].

### 3.5. Labor and Employment Practices

Various industries employ individuals in roles that involve them in violence. Private military contractors are a major international industry outside of the regulatory mechanisms of state-run militaries and the political and other pressures that constrain their involvement in violence. The role of mercenaries in human rights abuses including torture and extra-judiciary killings has been highlighted; their presence can also prolong conflicts and undermine peace efforts [78]. At an interpersonal level, staff in night-time environments deal with vulnerable or inebriated customers, and investment in their training can reduce violence, particularly when combined with community measures [79]. In multiple industries, the extent of bullying, sexual, and other workplace violence is hidden through non-disclosure agreements, the threat of legal repercussions, and intolerance of whistle blowers [80]. The nature of some industries such as the global pornography industry (valued at over USD 50 billion in 2022 [81]) can leave those choosing or forced to work in the industry routinely exposed to violence and feeling they have no recourse against their employers [82]. Moreover, many pornographic sites feature violence and abuse, particularly against women, with emerging evidence suggesting some behaviors may be mimicked by those watching them [83,84].

Many industries employ large labor forces to work with vulnerable people requiring care or containment, including in residential (e.g., care homes), prison, and healthcare settings. The relative proportions provided through state and private providers vary between nations. Regardless, patrons likely face lower risks of violence where evidenced-based practices, training, and monitoring are in place. Such practices can also protect staff against violent patrons. However, good practice can be compromised by profit motivation, with privately run prisons seeing poorer staff training and working conditions and higher rates of violence [85,86]. In some countries, care for victims of violence (e.g., child social care and sexual assault services) is increasingly being privatized, with profit prioritization risking standards of care and victims’ well-being [87,88]. 

Employment can reduce the risks of some forms of violence [89] and, by providing job opportunities, commercial actors may support violence prevention. Employment may also reduce recidivism in offenders [90]. Some companies have policies that welcome ex-prisoners [91]. Although relatively small in scale, some businesses advise on, develop, and implement programs aimed at reducing violence, which may increase awareness of violence and the capacity for prevention. Other commercial programs aim to support parents to reduce risks of child maltreatment and behavioral problems [92]. The extent of independent evidence on for-profit programs is contested, although they have stimulated the development of related non-profit interventions [93]. Whilst such programs helped establish the importance of parenting in reducing child abuse, quality parenting is impeded by commercial practices aligned with poor parental leave policies, low paid and insecure jobs, and excessive working hours. Poor working conditions disproportionately affect low-income workers, especially in low- and middle-income countries (LMICs), and lead to physical and mental ill-health [94]—factors associated with increased violence [95]. Activities to improve working conditions through unionization and union action can also be violently resisted by employers and state actors [96].

Wider considerations of commercial determinants describe ways in which commercial processes destabilize, outsource, and offshore production. Models that disinvest from areas when cheaper manufacturing locations become available can lead to mass unemployment events, with catastrophic impacts on communities including increased violence [97,98]. Although understudied, companies investing in redeployment and retraining staff may help prevent violence. Similarly understudied are impacts on violence from corporate practices that replace stable employment with unpredictable, poorer-paid, gig economy work. Such practices may impact mental health [99] and, consequently, violence. Company cost-cutting can also influence decisions to locate production in countries (or under flags of convenience) with poorer environmental and social protections, including weaker anti-violence legislation (e.g., on rape, slavery, firearms control, and legal representation for victims) [9]. In some cases, this can include companies’ support for forced labor [100]; use of child labor in settings with extreme physical, psychological, and social dangers [101]; and ethnic and other workforce discrimination [102]. Globally, one in five workers are estimated to have experienced workplace violence or harassment during their working lives [103], whilst millions of children face violence at work every day [104]. The combination of abusive working conditions and low pay can contribute to high-risk migratory practices, with inherent risks of violence. Low-paid employment can also contribute to financial and other inequalities between and within countries. Such inequalities have also been linked with increased risks of violence [105,106].

### 3.6. Financial Practices

Commercial behaviors may impact violence through choices on where to invest finances, profits, pensions, and other assets. Investigations into pension and other finance schemes have identified investments in arms trades, alcohol, and regimes with poor violence and human rights records [107,108]. Military industry stocks may even be favored investments during times of potential conflict. Violence exacerbating investment is not limited to private companies, with public sector financial instruments such as pensions having similar links [107]. 

Individuals with high, poorly managed debt are at increased risk of violence, including intimate partner violence [109]. Numerous commercial actors encourage debt through credit cards, mortgages, and loans which often allow continued spending by, and extract profit from, low-income individuals [11,110]. Such practices place stress on families [111], increasing risks of violence, including child maltreatment [112]. Where companies operate high-interest loans and debt collection services, these may trap people in cycles of poverty and stress with repercussions for violence. Violence may even be employed in debt collection, often through third-party agents. The risks of violence may be reduced when latitude is offered to individuals in debt, increasing their ability to retain homes, support children and other vulnerable individuals, and avoid involvement with unregulated illegal loan providers [113]. 

Governmental regulation and public scrutiny have historically been key levers to prevent companies from adopting the most profitable methods to produce and sell products. However, international choice of places to locate production, register taxation, and invest pensions and profits can enable commercial actors to exploit differences in regulation, legislation, and public scrutiny, leading to a ‘race to the bottom’ [114]. Financial incentives and concessions may be offered by or levered out of governments, including relaxed protections for workers, communities, and the environment, with potential repercussions for violence (Section 3.4 and Section 3.5). Financial sweeteners, tax concessions, and bailouts can also impact public funding, degrading tax income and public expenditure on social security and judicial measures that counter violence. Over a third of multi-national corporation profits (around USD 1 trillion) were estimated to be in tax havens in 2022, resulting in a 10% tax revenue loss globally [115]. Taxes paid are rarely scaled to include externalities such as the costs business may have on health, social, and environmental conditions [80].

Like other industries (Section 3.5), financial businesses may adopt ESG strategies; some financial companies and instruments now exclude industries and activities involving gambling, deforestation, and unfair labor practices [116]. This provides an opportunity for companies to move finances into investments that encourage violence prevention.

## 4. Discussion

We aimed to test a commercial determinants of health framework [11] through its application to violence. We have identified substantial overlap between the different aspects of commercial practice (Table 2). However, the segmented framework provides a way of deconstructing what is a complex set of relationships where commercial behaviors are often hidden behind offshore agreements, legal protections, and public-facing communications and reputational management activities. Examining this complexity from the perspective of violence captures industries already closely related to violence (e.g., firearms and alcohol), which, to some extent, are already considered from a commercial determinants perspective. However, it also reveals how the wider impacts of environmental management, employment conditions, financial investment, and choice of commercial and political partners can all work for or against violence. Effectively impacting commercial determinants in these areas requires a better understanding of the influences and options available to political and public sector actors. However, some themes are already apparent. 

Commercial determinants are more likely to promote violence where short-term profits are prioritized, especially when combined with short-sighted political support. Competitive markets and investors can favor disposable attitudes to resources, the environment, and even workforces. Longer-term externalities of pollution, poverty, and asset degradation may be disregarded by companies when they have no financial responsibility (e.g., taxation and fines) for the wider consequences of their actions. They may be equally disregarded by governments when business investments are at stake. As LMICs may provide weaker regulations and enforcement, their populations are often left with conditions more conducive to violence. The fact that LMICs see higher levels of violence globally is unlikely to be a coincidence [8]. Profit-at-any-cost models create an urgency for products to reach the market without sufficient consideration. Thus, repercussions on violence are only identified post-launch. Such behaviors contradict guiding principles for business and human rights, which call for precautionary approaches to environmental challenges, environmentally friendly technologies, and the elimination of forced, compulsory, and child labor and discrimination [117]. Like other voluntary codes of business practice, they are largely ineffectual at changing commercial behaviors. However, they still appear in portfolios of initiatives quoted for reputational management, potentially subverting calls for effective regulation. 

Links between commercial behaviors and violence are not new. From the beginnings of global capitalism, corporations have contributed to problems like violence, labor exploitation, and environmental degradation [118]. This is a reason why their activities have been regulated by governments seeking to drive more equitable, less harmful patterns of trade and manufacturing. Despite power having recently slipped away from governments, largely to multi-national corporations, governments can still regulate and legislate, especially when working internationally. They should be accountable for demonstrating the use of tax income and other financial assets to improve commercial behaviors. For instance, using figures showing 70–90% of guns recovered from crimes scenes in Mexico are trafficked from the USA, the Mexican government have brought a lawsuit against US gun manufacturers and one distributor alleging they make deliberate design, marketing, and distribution choices to retain and grow that illegal market [119].

Investment should support academia, public health, and social sectors and ensure an independent media exposing profiteering through business models that support violence directly or indirectly create or invest in working conditions and regimes conducive to violence. Public exposure of poor business practices may be one of the most promising areas to influence commercial behaviors. Complex, speculatively funded business models often depend on the trust that people place in them. Changes in trust can abruptly alter commercial value, investment, and media and political support [120]. Businesses such as sport, music, and tourism are equally exposed to the tides of public preference and greater exposure of their role in violence may affect change. Political and public interest in connections between commerce and violence may drive similar interest in boardrooms but needs to result in real change rather than further reputational management.

Although others have recognized inherent risks in discussing the benefits of commerce (e.g., detracting attention from continuing harmful behaviors), most commercial entities are neither entirely good nor bad [4]. Employment has already been linked with lower levels of domestic violence and child abuse [89], although poor pay and working conditions may be linked with increases. In peace economics, international trade has been linked with reduced risks of conflict as a result of increased cooperation between countries [121]. Equally, corporate philanthropy can support violence prevention research and interventions even if its intention is to improve companies’ competitive edge, reputation, and, consequently, profits. Genuine commercial altruism, where companies undertake activities to benefit society at their own significant expense, seems inconsistent with contemporary neoliberal business models. However, companies can work with governments towards a level playing field for industry that rewards commercial practices known to reduce violence. Providers of services to children and other vulnerable individuals can lobby to improve their patrons’ protection from violence rather than cut protection to increase profits. Even commercial areas that rely on the threat of violence to prosper (e.g., arms and security) can adopt violence prevention approaches, such as limiting the distribution and public advertising of their products; building in security measures to control misuse; protecting distribution and storage; and ensuring new technologies minimize unlicensed use and risks to unintended targets. Where companies seek to operate in settings currently suffering from violence, lobbying for and negotiating progressive policy and legislation reforms should be a commercial expectation rather than seeking fiscal benefits and cheap labor. To accelerate change, a virtuous circle is needed that exposes and publicizes genuine good practice, helping reward it with more investment and a stronger customer base.

International agencies are already considering how models that better reflect health, peace, and sustainability could replace ones fixated on profit and GDP [122,123]. The Sustainable Development Goals provide another opportunity to understand how reductions in violence can be delivered alongside decent work, economic growth, and action on climate change and pollution. Whilst sustainability has arguably already penetrated boardroom discussions, violence prevention urgently needs political and public advocacy. In particular, misconceptions that violence arises randomly or is simply the product of particular individuals or dictators should be replaced with the recognition that violence arises when some individuals are left destitute whilst others are opulent; when people feel discriminated against and without a voice in their own fates; or when the state is not seen to protect families, communities, and belongings [8,124]. Such protection includes that from commercial exploitation. Both protection of and respect for the values of indigenous people are likely to result in less bellicose and more sustainable commercial ventures. Moreover, the need for protection could be moderated if established tools such as health impact assessments [125] are used to identify harms expected to result from investments, developments, and practices (e.g., social media advertising algorithms promoting extremism) in advance of their launch [126]. 

Currently, the race between commercial competitors to dominate emerging markets frequently works against proper consideration of the health and social consequences of new developments, with precautionary principles diluted or outright dismissed. However, we are in a period of unprecedented change. Artificial intelligence (AI) is likely to touch all aspects of life and other technological developments mean companies can both monitor and predict individuals’ behavior with much greater precision. With such tools increasingly available, commercial players have ample ability to predict the impacts of their plans and their pipeline products on violence in a timely fashion. For instance, vehicles are commonly weaponized and deployed to deadly effect [127] and developments in assisted driverless technologies should actively exclude their use for violence. Equally, virtual reality does not need to repeat the mistakes previous IT platforms have made communicating, even advertising, violence, terrorism, and other hateful and incendiary content. There is potential for AI to be developed and deployed to identify and reduce violence propagated online. Many opportunities to change commercial behaviors in order to reduce violence are already apparent and further independent research should establish a broader evidence base for violence prevention across each of the commercial practices (Table 2) examined here. 

## 5. Conclusions

Applying Gilmore and colleagues’ [11] framework of commercial practice, it is irrefutable that commercial power is used in ways that contribute to violence. Recognizing commercial determinants of violence adds to claims that “the cumulative effects of commercial activity are arguably the greatest threat to human and planetary health of the 21st century” [128]. Action is required urgently. Opportunities to address this threat are diminishing as wealth and power slip from democratic governmental bodies to commercial entities answerable largely to self-appointed boards and shareholders. Diminished independent media means dominant narratives are increasingly aligned with commercial interests—describing interventions which may beneficially alter commercial determinants as ‘nanny state’ and ignoring how children and adults are increasingly educated in environments designed by corporations to encourage the unbridled pursuit of profit.

Commercial organizations have much to gain from peaceful and, consequently, more prosperous societies. The return of war to Europe and escalations in conflict in the Middle East are stark reminders of the negative impacts of violence on communities and commerce. Generally, elevated violence within and between nations drastically reduces opportunities for growth and prosperity. By contrast, the development of business in countries affected by fragility, conflict, and violence may act to stabilize regions [129]. Preventing violence, like climate change, may look like an insurmountable challenge but the causes of both are predictable and amenable to change. People who feel they have lost everything are more likely to act like they have nothing to lose. Ensuring fewer people find themselves in such situations requires moving from the dogged pursuit of commercial profit to models that recognize money linked with violence will ultimately end in a poorer world for everyone.

## Figures and Tables

**Table 1:** Framework from Gilmore et al. of commercial sector practices that may harm health [11].

Practice	Definition
Political	Practices to secure preferential treatment or prevent, shape, circumvent, or undermine public policies in ways that further corporate interests
Scientific	Practices involving the production and use of science to alter products or otherwise secure favorable outcomes (or both) for the industry
Marketing	Practices to promote sales of products or services
Reputational	Efforts to shape legitimacy and credulity, reduce risk, and enhance corporate brand image
Supply chain and waste	Practices involved in the creation, distribution, retail, and waste management of products or services
Labor and employment	Practices to manage people employed directly within, or under contract to, the organizations within its supply chain
Financial	Practices to support the financial position of the organization

**Table 2:** Examples of, and associations between, risks of violence relating to different aspects of commercial practice.

Practice						Example
●						Lobbying against violence-reducing policies and restrictions (e.g., alcohol and firearms control)
●			●			Resisting implementation of environmental protections (e.g., leading to deterioration of local resources and competition for food, water, etc.)
●	●	●				Influencing scientific balance by funding distorting research (e.g., through individuals, universities, and governments)
	●	●				Influencing scientific opinion (e.g., undermining evidence, individual studies, and academics)
	●	●		●		Using judicial mechanisms to block individuals or media exposing corporate involvement in violence and violence-related practices
	●	●				Using commercial investment to influence media coverage of scientific evidence (e.g., newspapers and quasi scientific magazines)
	●	●				Influencing the content of educational programs to obscure effective practices that could prevent violence (e.g., alcohol education)
		●				Marketing violence-related products at children (e.g., firearms and alcohol)
		●				Using popular prosocial brands to deflect attention from violent regimes and practices (e.g., sports washing)
		●				Making marginal philanthropic investments to deflect focus from wider violence-enabling activities
			●			Intimidation of local communities resisting commercial developments (e.g., mining and deforestation)
			●			Producing pollutants directly related to increased violence risks (e.g., lead and air pollution)
			●			Degrading local access to food, clean water, and other essential resources
			●			Contributing to violence related to climate change (e.g., increased temperature linked with violence)
●			●	●		Locating commercial activities in countries with poor violence legislation, regulation, and human rights
●			●		●	Locating commercial assets, profits, and financial activities in countries with poor environmental credentials
●			●			Utilizing maritime flags of convenience to avoid legislation that can impact violence
●				●		Inadequate employee training and regulatory practices in industries relating to violence (e.g., door staff and private military)
				●		Not implementing protective regulation in the workplace (e.g., against workplace bullying, slavery, discrimination, and child labor)
				●		Poor internal company policies towards violence prevention and the protection of vulnerable staff and consumers
				●		Fueling population migration with associated risks of violence through poor employment conditions, discrimination, and abuse
●				●	●	Cost-cutting for profit in private social industries (e.g., prison, school, health, and care settings) which reduces violence protection and victim care
				●	●	Contributing to mass unemployment events through unmitigated disinvestment, increasing risks of violence
					●	Encouraging unmanageable debt in consumers and aggressively pursuing debt collection
					●	Investing corporate profits, pensions, and other finances in industries, financial mechanisms, or regimes connected with violence and its causes

Key: ● Political ● Scientific ● Marketing and reputational ● Supply chain and waste ● Labor and market ● Financial.

## Data Availability

No new data were created or analyzed in this study. Data sharing is not applicable to this article.
